# Web-Based Quality Assurance Process Drives Improvements in Obstetric Ultrasound in 5 Low- and Middle-Income Countries

**DOI:** 10.9745/GHSP-D-16-00156

**Published:** 2016-12-23

**Authors:** Jonathan O Swanson, David Plotner, Holly L Franklin, David L Swanson, Victor Lokomba Bolamba, Adrien Lokangaka, Irma Sayury Pineda, Lester Figueroa, Ana Garces, David Muyodi, Fabian Esamai, Nancy Kanaiza, Waseem Mirza, Farnaz Naqvi, Sarah Saleem, Musaku Mwenechanya, Melody Chiwila, Dorothy Hamsumonde, Elizabeth M McClure, Robert L Goldenberg, Robert O Nathan

**Affiliations:** a University of Washington Medical Center, Seattle, WA, USA.; b RTI International, Durham, NC, USA.; c University of Kinshasa, Kinshasa, Democratic Republic of the Congo.; d Instituto de Nutrición de Centroamérica y Panamá (INCAP), Guatemala City, Guatemala.; e Moi University School of Medicine, Eldoret, Kenya.; f Aga Khan University, Karachi, Pakistan.; g University Teaching Hospital, Lusaka, Zambia.; h Columbia University, New York, NY, USA.

## Abstract

High quality is important in medical imaging, yet in many geographic areas, highly skilled sonographers are in short supply. Advances in Internet capacity along with the development of reliable portable ultrasounds have created an opportunity to provide centralized remote quality assurance (QA) for ultrasound exams performed at rural sites worldwide. We sought to harness these advances by developing a web-based tool to facilitate QA activities for newly trained sonographers who were taking part in a cluster randomized trial investigating the role of limited obstetric ultrasound to improve pregnancy outcomes in 5 low- and middle-income countries. We were challenged by connectivity issues, by country-specific needs for website usability, and by the overall need for a high-throughput system. After systematically addressing these needs, the resulting QA website helped drive ultrasound quality improvement across all 5 countries. It now offers the potential for adoption by future ultrasound- or imaging-based global health initiatives.

## Abstract (precis)

Newly trained sonographers improved performance through a quality assurance process that merged (1) evaluation by remote experts of images uploaded to a website, with (2) periodic in-person skill tests. To promote sustainability, in-country supervisors gradually assumed more responsibility for image evaluation. The user-friendly and efficient interface used simple menus and forms, customized based on the user's role.

## BACKGROUND

Obstetric ultrasound is an important tool for determining gestational age and also for identifying medical conditions during pregnancy that, if addressed, can lead to improved maternal and fetal outcomes.^1^^–^^9^ Although the use of ultrasound is increasing in low- and middle-income countries, an important barrier to widespread implementation is the lack of trained sonographers. Furthermore, even when training is available, strategies for retention of skills are needed. Especially in rural settings, the availability of adequate oversight following training, including review of diagnostic accuracy and other quality assurance (QA) activities for newly trained health care professionals, is limited and frequently nonexistent.

Skilled sonographers are in short supply in many low- and middle-income countries.

However, technology, including increasing Internet capacity, can facilitate remote support of health care providers.^10^^–^^12^ Advances in communication technologies along with the development of reliable portable ultrasounds have created the opportunity to provide remote QA to rural sonography sites across the globe. Given this context, we sought to develop a web-based tool to facilitate QA activities for newly trained sonographers.

This QA project was undertaken as part of the First Look trial of obstetric ultrasound to improve pregnancy outcomes. First Look was conducted by the *Eunice Kennedy Shriver* National Institute of Child Health and Human Development's (NICHD's) Global Network for Women's and Children's Health Research (Global Network) at sites in the Democratic Republic of the Congo (DRC), Guatemala, Kenya, Pakistan, and Zambia. RTI International served as the data coordinating center.^13^ Each study site employed both a centrally located supervising sonographer and several field sonographers who were newly trained for the study.

For First Look, the University of Washington (UW) Department of Radiology created a training course in screening obstetric ultrasound. The goal was for health care professionals with no prior ultrasound experience (e.g., midwives, nurses, clinical officers, radiographers, and medical officers) to screen for pregnancy complications as part of the clinical trial.^14^ The resulting training course differed significantly from traditional general diagnostic ultrasound training, which can span 3–4 years with built-in oversight and QA during the extended curriculum. In contrast, the First Look training curriculum was designed to avoid the opportunity costs to individuals and communities that occur when health care workers leave rural communities for extended training. The hands-on course was run by the QA team, which consisted of UW radiologists and/or sonographers in collaboration with the experienced sonographer at each country site, who then served as the in-country QA reviewer. The training consisted of a 2-week intensive course in basic obstetric ultrasound and a 12-week pilot phase in which trainees performed basic ultrasound exams on pregnant patients who presented to intervention health centers.

This project aimed to train health care workers with no previous ultrasound experience to screen for pregnancy complications.

The 2-week basic obstetric ultrasound course, under the supervision of the UW Department of Radiology, was administered to 41 ultrasound-naive health care workers at 5 sites: Karawa, DRC; Chimaltenango, Guatemala; Eldoret, Kenya; Karachi, Pakistan; and Lusaka, Zambia. The course was group-based and the curriculum was modified slightly for each country. Competency was assessed by written tests at the conclusion of the 2-week hands-on portion of the training. In addition, there was a timed scanning skills test at the conclusion of the course as well as after the 12-week pilot phase of the study. The results and success of this training are described in greater detail in a separate article currently in press at the time of this publication. Following the intensive 2-week hands-on training experience at each site, the 41 field sonographers then proceeded to conduct ultrasound examinations as part of antenatal care in health centers, with oversight by their in-country supervising sonographer and the UW, during the study's 3-month pilot phase and then throughout the 18-month trial period, which concluded in August 2016.

Following 2 weeks of hands-on training, 41 field sonographers conducted ultrasound exams as part of antenatal care.

During the pilot phase, every case (3,800 exams in total) was reviewed and rated by the off-site QA reviewers. After the pilot phase, 10%–20% of all study exams (an estimated 5,000 additional exams) were reviewed for the trial. The reviews were performed by radiologists based in the United States and by the in-country supervising sonographers, who were often based at a central academic center several hours or more away from the field sites where the ultrasounds were performed. Thus the review process required an efficient, effective, and user-friendly web-based QA system. We sought to provide the ability for experts, at the UW as well as at each study site, to review and provide rapid and specific feedback on ultrasound images to field sonographers working in remote regions. The Global Network ultrasound website was thus created to be the central hub for transmitting locally collected data to reviewing radiologists and sonographers, and for the in-country QA sonographers to target remedial training for the new sonographers. The primary objective of the website was to facilitate QA activities for the obstetric ultrasound scans.

Experts provided rapid and specific feedback on images to the field sonographers working in remote regions.

The UW radiology team designated which images were required to be acquired and saved for each patient imaged—placental position, placental fluid evaluation, fetal position, cervix (with length, if applicable), and biometry measurements—and developed criteria for assessing the quality of submitted images. RTI International created the website and data management process and provided overall logistical coordination.

Funding for development of the First Look QA website was provided through grants from the Bill & Melinda Gates Foundation to RTI International and through support from the NICHD, with additional funding from GE Healthcare to support the UW activities. The study used the research infrastructure for and was carried out under the direction of the NICHD-funded Global Network for Women's and Children's Health Research.

## INTERVENTION: WEBSITE AS QA TOOL

The prototype for the web-based QA system was developed by RTI International's Data Coordinating and Analysis Center for the ongoing Nulliparous Pregnancy Outcomes Study: Monitoring Mothers-to-Be (nuMoM2b). nuMoM2b is an observational study, funded by NICHD, of approximately 10,000 women residing in the United States who are followed longitudinally through their first pregnancy.^15^ The original web application was created to facilitate a required central ultrasound credentialing process for cervical length measurement, uterine artery Doppler assessment, and fetal adrenal gland measurements. For the nuMoM2b study, authorized staff from the participating clinical sites upload deidentified images, measurements, and sonographer identification information through the study website. These data are managed by the web application, and each set of images and measurements is assigned to an appropriate expert certifier for review. The certifier is automatically alerted by email of a new applicant and views submission materials from the website. Results of the review are posted back to the website by the expert certifier for access by the coordinator at the local clinical site.

For the First Look ultrasound trial, the adapted QA website allowed staff at study sites to upload sonography exam data for review and evaluation. The data (images, report, and brief assessment) were deidentified but remained associated with the field sonographer who performed the ultrasound exam. An expert was then able to log in to the password-protected website to review and provide comments on the images. The website featured individual accounts and role-based security. The key roles in using the system included administrators (who had the ability to create and manage accounts), data center coordinators (responsible for uploading images for review), supervising sonographers (for in-country QA review), and UW-based QA reviewers.

### Image Creation and Transfer

As discussed previously, a team of developers at RTI International and radiologists at UW designed the QA system around the study expectations and prescribed images for each ultrasound exam. For second- and third-trimester exams, the field sonographer obtained images that demonstrated fetal number, fetal position, placenta position, cervical length (when appropriate), biometry measurements, and amniotic fluid volume. For a single-gestation pregnancy, the prescribed set of images included a midline sagittal image of the cervix (with measurement if earlier than 26 weeks), standard fetal biometric measurements (biparietal diameter, head circumference, abdominal circumference, and femur length), 1–4 images to measure amniotic fluid volume (for measurement of maximum vertical pocket or the amniotic fluid index, depending on gestational age), an image showing location of placenta, and an image demonstrating lie of fetus. For twin pregnancies, in addition to the above-mentioned images, the study required fetal position documentation, biometric measurements for each fetus, and an image documenting chorionic/amniotic arrangement of the twin pregnancy. For each first-trimester exam, the field sonographers provided images documenting mean sac diameter or crown rump length. For each image, the QA team evaluated ultrasound technique in terms of zoom, depth, and focus, as well as measurement technique for biometry.

For each image, the QA team evaluated ultrasound technique in terms of zoom, depth, focus, and measurement technique.

All field sites used the LOGIQ e Ultrasound BT12 for the First Look trial.^13^ The field sonographers used the LOGIQ e built-in obstetric summary sheet as a skeleton report to document biometry, including estimated dates and fetal weight, fetal presentation, and twin pregnancy. The field sonographers used the LOGIQ e anatomy worksheet to document impressions and next steps succinctly. For example, for a mother whose exam demonstrated a late third-trimester breech pregnancy or twins, the field sonographer documented that the patient was being appropriately referred. These worksheets were then saved as JPEG images and were included in the standard set of images that were centrally reviewed for each exam.

### Image Acquisition and Interpretation

The website was designed to require the fewest intermediate steps when delivering information from the ultrasound machine to the reviewer. The process involved an express examination export that created an easily accessible folder of JPEG images on the ultrasound hard drive with the exam identification number and the examination date as part of the folder name.

The website was designed to transfer information from ultrasound machine to QA reviewer in the fewest steps possible.

**FIGURE 1:**
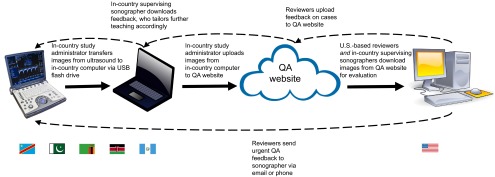
Diagram of the Web-Based Obstetric Ultrasound Quality Assurance Process in 5 Low- and Middle-Income Countries^a^ Abbreviation: QA, quality assurance. ^a^ Democratic Republic of the Congo, Guatemala, Kenya, Pakistan, and Zambia.

An in-country study administrator transferred the images from the ultrasound machine to a secure, centrally located, in-country computer that had access to the Internet (Figure 1). Whenever possible, but at a minimum on a weekly basis, the same in-country study administrator uploaded the ultrasound images to the website in a compressed format (see below for discussion of connectivity and bandwidth challenges). In general, the QA system was not used to triage emergent cases: Although cases could be flagged on the website for emergent review, it was much more common for field sonographers to relay patient-deidentified imaging findings via smartphone to the supervising sonographer or to the referral sonographers at the referral hospital, for real-time consult and triage of potentially emergent cases.

The exams to be reviewed were then made available to the relevant in-country supervising sonographer and to the UW radiologists, who could reference specific cases for further instruction. The email function embedded into the QA website allowed for more rapid communication between reviewers and field sonographers, which proved useful especially in cases where a reviewer's comments had the potential to change clinical management.

### Quality Review Platform

**FIGURE 2:**
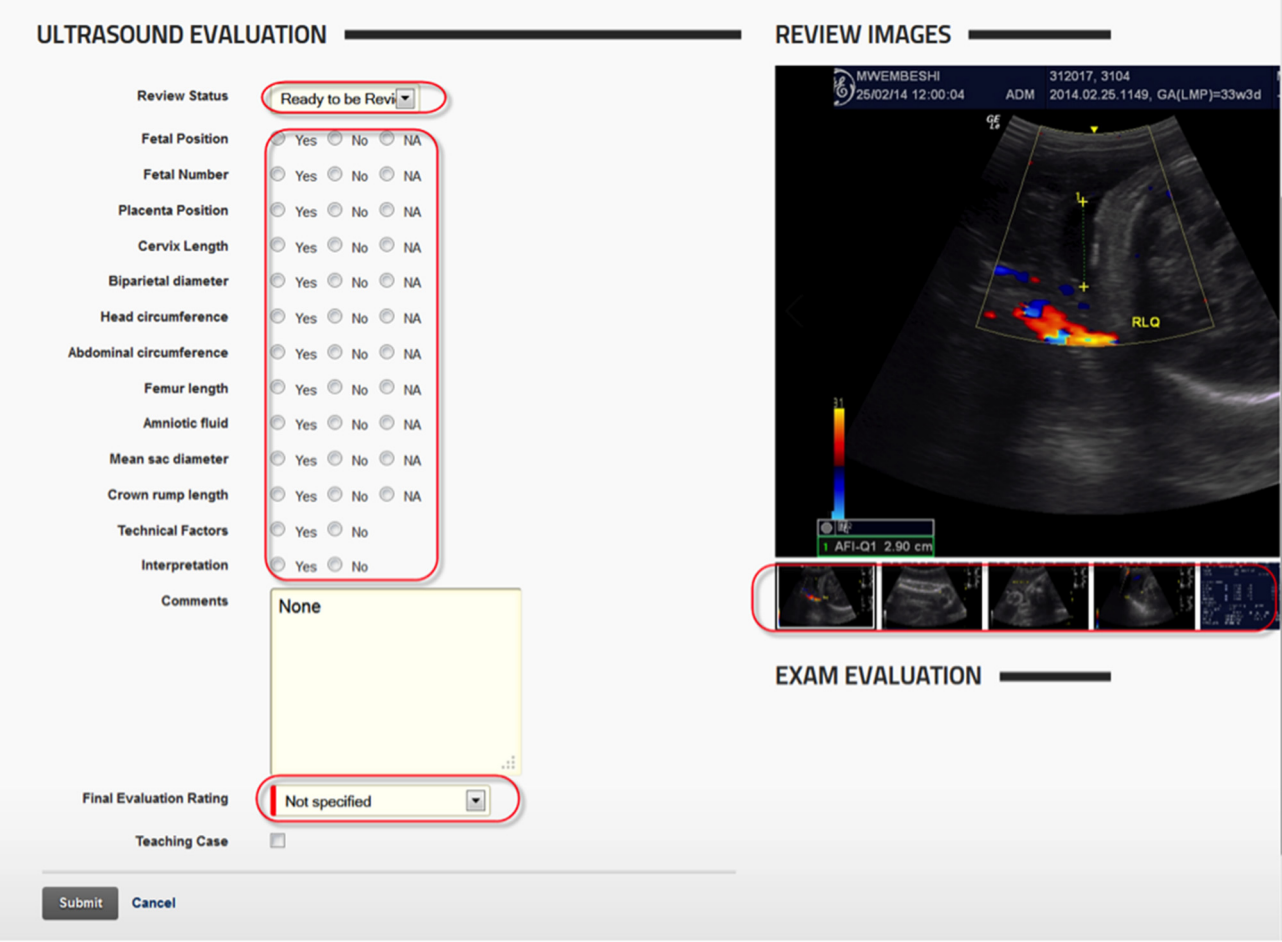
Screen Shot of the Evaluation Checklist From the Ultrasound Quality Assurance Website The reviewer's simple checklist is on the left with radio buttons to indicate either "yes" for high quality, "no" for poor quality, or "NA" (not applicable). Below, there is room for comments, a drop-down menu for the final evaluation decision, and a checkbox to indicate potential as a teaching case. The images are on the right, with easy-to-navigate scrollable thumbnail images below the selected image.

The review section of the website (Figure 2) was designed for rapid evaluation of the image captures and reports for each patient. The prescribed image sets were available in an easy-to-use image slider embedded next to the evaluation form, which asked reviewers to select radio-button options to record quality. With this system, cases could be reviewed in 3–5 minutes. All reviewers used standardized rating criteria and comments. Spanish and French translations of the reviews were provided for sonographers in Guatemala and the DRC, respectively. (English is an official language in Kenya, Pakistan, and Zambia.) Reviews were generally completed within 1–2 weeks of a standard, nonurgent screening ultrasound exam being performed. The UW radiology team reviewed about 75% of the 3,800 exams evaluated during the 12-week pilot training phase, while the in-country supervising sonographers performed approximately 25% of these QA reviews.

Within the QA website, cases could be reviewed in 3–5 minutes.

Multiple technical criteria were assessed and scored, as well as the final interpretation, and a final evaluation of acceptable, suboptimal but acceptable, or unsatisfactory was assigned based on the scoring of the exam.

After reviews were completed, the exams with ratings and comments were available on the website for the local trainers and trainees to view. Interesting cases (such as classic appearance of rare finding such as placenta previa), cases demonstrating common mistakes, and cases demonstrating superb technique were marked as teaching cases, and these teaching cases were shared across all 5 country sites.

QA reviewers identified teaching cases, which were shared across all 5 country sites.

### Feedback to Field Sonographers

The QA review created a dynamic feedback loop between the reviewers and the field sonographers. The in-country supervising sonographers met with the field sonographers at least once every 2 weeks to discuss the QA reviews that had been completed. The supervising sonographers could use a computer to display cases from the website or could bring the identifying numbers for specific cases to health centers and pull the cases up on the field sonographer's ultrasound machine. For errors requiring more immediate attention, the QA reviewers at UW emailed the in-country supervising sonographers through the QA website.

In addition to reviewing exams on the QA website, the trainers observed each trainee scanning at an intervention health center at least once every 2 weeks during the pilot phase. Conference calls between UW radiologists and the in-country supervising sonographers took place periodically during each site's pilot phase to discuss each trainee's progress. These conversations incorporated the scan reviews as well as the local trainers' direct observations. They also allowed the UW radiologists to share best practices from other study sites that might be relevant to QA issues. The data on the website generated from the reviews helped the local trainers target remedial training for specific trainees, as well as demonstrated larger trends from which all the field sonographers could learn. Ultimately, the feedback from the website review was merged with periodic in-person scanning skill tests.

## RESULTS OF THE WEB-BASED QA SYSTEM

### Benefits

#### Ultrasound Quality Improvement

The QA website facilitated effective and timely communication between the U.S.-based reviewers at UW, the in-country sonographers who were also QA reviewers, and the field-based sonographers at the study sites. The exams that were reviewed made it possible for reviewers to target where remedial training was needed. The in-country supervising sonographers could also use the website to generate examples of proper and improper technique as well as to find illustrative teaching cases, which were labeled during the reviews.

The exams that were reviewed allowed local trainers to target where remedial training was needed.

As described previously, final evaluation of an exam as acceptable, suboptimal but acceptable, or unsatisfactory was determined based on the reviewer-assigned scores. In the first month of the pilot phase, 21.5% of exams at the sites, on average, were rated unsatisfactory. In the third and final month of the pilot phase 10.0% of exams at the sites, on average, were rated unsatisfactory. Identification of errors was strictly adhered to so that, for instance, wrong biparietal diameter level or slightly inaccurate caliper placement on the calvarium both resulted in unsatisfactory ratings. That being said, errors in the third month tended to be minor, such as slightly inaccurate caliper placement rather than wrong anatomic level.

Exams were marked acceptable, suboptimal but acceptable, or unsatisfactory based on reviewer-assigned scores.

The trend in error rates was consistent with the training design. The initial hands-on training occurred over a short 2-week period. The trainees then returned to their health centers and began the 12-week pilot phase, which was designed to provide the trainees with the necessary additional hands-on experience, as well as on-site training, to improve the quality of their exams. The overall improvement over time speaks to the success of the remote QA system helping to target teaching of specific imaging deficiencies to specific field sonographers.

In addition, we would like to point out that the unsatisfactory rates noted above underestimate the overall quality of the scanning. The QA grading system was designed to deem a study unsatisfactory if any of the single images that demonstrated required scanning components (fetal position, fetal number, placental position, cervical length, biparietal diameter, head circumference, abdominal circumference, femur length, amniotic fluid assessment, and technical factors) was technically deficient. The goal of the tight ratings was to provide teaching opportunities for the field sonographer. However, a better assessment of scanning proficiency is agreement per image and agreement in final ultrasound diagnosis. In agreement per image, the reviewers rated 94.8% of the images obtained by the field sonographers satisfactory, and there was a 99.4% concordance between the field sonographers and the reviewers in the final ultrasound diagnosis.

#### In-Country Ownership and Capacity

Incorporating the in-country supervising sonographer as a QA reviewer as well as the in-country conduit for distributing feedback from the website reviews to the field sonographers proved successful on multiple fronts. The supervising sonographers translated the QA system and approach into the varied conditions existing in their own country. Each country's supervising sonographer assisted in the initial 2-week training and had access to their field sonographers' common mistakes, as highlighted by the U.S.-based reviewers. In the case of attrition of field sonographers, the supervising sonographer led the training of replacement sonographers, using existing QA feedback found on the website to tailor such trainings to address common mistakes. In addition, the supervising sonographers did an increasing number of the QA reviews as the pilot phase and trial progressed; this was consistent with the study objective and with the study's goal of developing an in-country sense of ownership over the process. The QA website thus facilitated the transition of QA review to in-country reviewers, while still allowing central review across the 5 sites to ensure consistency of data. All of these activities increased the likelihood of program sustainability independent of the U.S.-based reviewers.

The web-based QA system facilitated the transition of QA review from U.S.-based to in-country reviewers.

### Challenges

#### Connectivity Challenges

Some of the participating health centers had limited, semi-reliable access to the Internet, and with approximately 750 cases being uploaded from each site over the 12-week pilot period, we needed an efficient upload strategy. In the end, this pivoted around 2 variables: image file formats and file management.

- **Image file formats:** The LOGIQ e was able to export exam data in either DICOM-2 or JPEG formats. Although DICOM-2 would have allowed more comprehensive exam information to be shared, it was deemed unnecessary, as all the relevant information could be captured in exam screens and image captures with 7–10 JPEG files per exam. JPEG files were much smaller, which reduced concerns over bandwidth availability for uploads at the data centers
- **Use of zipped exam files:** Site coordinators were able to zip exam folders into a single file and upload the compressed files in a single upload. This simplified the process, while reducing bandwidth and upload errors. The website automatically unzipped the files on upload and made the individual files available to the reviewers.

#### Usability Challenges

The website was used by individuals with varying computer adroitness and different native languages and roles in the project. To be effective, the website had to interact appropriately with all of these users. One of the most important steps to achieving this goal was partitioning the site functions by study role (i.e., QA by supervising sonographers vs. input by field sonographers). Designing role-based access to the website allowed for simplified menus and options customized for each user. Because each user saw only the options pertinent to their task, site navigation and use went faster, regardless of the user's role.

The QA website was used by individuals with different roles, languages, and levels of computer proficiency.

#### Throughput Challenges

With nearly 4,000 cases reviewed during the 3-month pilot phase and another 5,000-plus cases reviewed during the trial, the QA reviewer needed an efficient way to access exams and provide consolidated feedback and ratings. We thus created a single interface to evaluate exams, regardless of gestational age. The interface consisted of a review sheet with a series of radio buttons and drop-down menus. Also on the page was an image slider that showed all of the exam images, allowing the reviewer to quickly select each image to evaluate the various attributes of the exam.

Acquiring and sending ultrasound "sweeps" or cine clips is an alternative method that has been used to review and analyze ultrasounds in the global health setting.^16^ Given the sheer number of exams that we needed to review, we posited assigning prescribed representative images as a faster, more efficient method. This method also allowed reviewers to provide each sonographer with clear feedback associated with specific images, diagnoses, and clinical scenarios.

### Feasibility

To create a QA system that users would find feasible, we had to both develop a centralized QA website and ensure that proper hardware was available and functioning at the in-country sites. There was also the issue of the hours needed to perform the QA. In the First Looks study's pilot phase, while field sonographers were being trained, approximately 50 working hours were needed to perform the QA review for each of the 5 study sites (4 minutes per case for 750 cases per site). The transferring and uploading of cases accounted for another 24 hours of work per country during this 12-week pilot phase. To limit the burden on local health care providers, the uploading was performed by a member of the study's in-country administrative staff. As we transitioned into the trial phase, the QA review became less intense because we reviewed only 10% to 20% of the cases performed.

### Lessons Learned

The ideal QA website needs to balance information and bandwidth usage. Time requirements and other difficulties in uploading images or in rendering pages with large images have to be balanced against providing enough information for an effective and rapid evaluation.

The ideal QA website needs to balance information and bandwidth usage.

## CONCLUSIONS

The review website and its features made it possible for a team of experts and training staff to provide rapid evaluation and feedback to newly trained sonographers working in several countries simultaneously. This web-based QA system facilitated direct feedback to training sonographers on cases they scanned unobserved, and tracking of sonographers' progress in skills during the period of the study.

The review process developed for the First Look study was tailored to a specific ultrasound machine, for ease of image transfer. However, the process itself is generic enough that with minor changes in guidance for the data export process, it should be effective with any ultrasound machine capable of exporting exam image files. Modifications to the process for transferring images to a website need to be sensitive to bandwidth availability at the remote sites that are to be evaluated.

We are planning to expand this website to include cloud-based hosting and modifications that will allow more efficient image upload and rendering. These improvements are expected to result in a faster-performing site, which will allow all users of the system to process more exams within a given time frame, even with poor Internet connectivity. We also expect this expansion of the website to allow us to work with field sites that use different ultrasound machines, and the QA process will be further refined to simplify any associated implementation challenges.

We plan to expand the website to be able to process more exams more quickly, even with poor Internet connectivity.

In developing a web-based tool to facilitate QA activity for this study, we were challenged by connectivity issues, by country-specific needs for website usability, and by the overall need for a high-throughput system. After systematically addressing these needs, the resulting QA website helped drive ultrasound quality improvement across all 5 countries. It now offers the potential for adoption by future ultrasound- or imaging-based global health initiatives.

## Notes

**Peer Reviewed**

**Cite this article as:** Swanson JO, Plotner D, Franklin HL, Swanson DL, Lokomba Bolamba V, Lokangaka A, Sayury Pineda I, Figueroa L, Garces A, Muyodi D, Esamai F, Kanaiza N, Mirza W, Naqvi F, Saleem S, Mwenechanya M, Chiwila M, Hamsumonde D, McClure EM, Goldenberg RL, Nathan RO. Web-based quality assurance process drives improvements in obstetric ultrasound in 5 low- and middle-income countries. Glob Health Sci Pract. 2016;4(4):675–683. http://dx.doi.org/10.9745/GHSP-D-16-00156
